# Engineering a dirhodium artificial metalloenzyme for selective olefin cyclopropanation

**DOI:** 10.1038/ncomms8789

**Published:** 2015-07-24

**Authors:** Poonam Srivastava, Hao Yang, Ken Ellis-Guardiola, Jared C. Lewis

**Affiliations:** 1 Coskata Inc. 4575 Weaver Parkway, Warrenville, IL 60555, USA; 2 Department of Chemistry, University of Chicago 5735 S. Ellis Avenue, Chicago, IL 60637, USA

## Abstract

Artificial metalloenzymes (ArMs) formed by incorporating synthetic metal catalysts into protein scaffolds have the potential to impart to chemical reactions selectivity that would be difficult to achieve using metal catalysts alone. In this work, we covalently link an alkyne-substituted dirhodium catalyst to a prolyl oligopeptidase containing a genetically encoded L-4-azidophenylalanine residue to create an ArM that catalyses olefin cyclopropanation. Scaffold mutagenesis is then used to improve the enantioselectivity of this reaction, and cyclopropanation of a range of styrenes and donor–acceptor carbene precursors were accepted. The ArM reduces the formation of byproducts, including those resulting from the reaction of dirhodium–carbene intermediates with water. This shows that an ArM can improve the substrate specificity of a catalyst and, for the first time, the water tolerance of a metal-catalysed reaction. Given the diversity of reactions catalysed by dirhodium complexes, we anticipate that dirhodium ArMs will provide many unique opportunities for selective catalysis.

## Abstract (web summary)

Artificial metalloenzymes (ArMs) have the potential to improve transition metal reactivity in complex media. Here, the authors link a dirhodium catalyst to a prolyl oligopeptidase to create an ArM that catalyzes enantioselective olefin cyclopropanation in aqueous solution.

## Graphical abstract

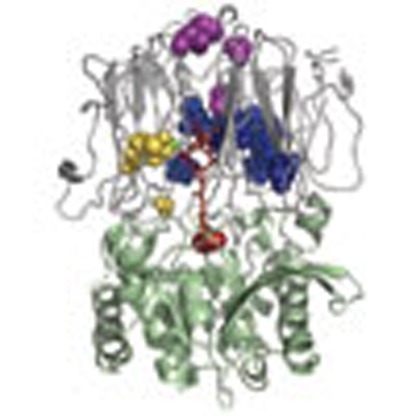

New approaches to control the selectivity and specificity of catalysts remain the subject of intense academic and industrial research because of the importance of selective catalysis for the synthesis of chemicals ranging from fuels to pharmaceuticals^1^. Weak interactions between substrates and catalysts imparted by functional groups distal to catalyst-active sites^2^,^3^ and supramolecular catalyst scaffolds^4^,^5^ are increasingly used to improve catalyst selectivity. Of course, such features are ubiquitous in enzymes^6^ and contribute to their often stunning activities and selectivities. To exploit the substrate-binding and activation capabilities of enzymes for reactions and catalysts not found in nature, researchers have developed a range of methods to link synthetic catalysts and protein scaffolds to create artificial metalloenzymes (ArMs)^1^,^7^. These efforts have culminated in ArMs for enantioselective, regioselective and chemoselective reactions, but, despite several notable examples^8^,^9^,^10^,^11^,^12^,^13^, engineering scaffolds to further improve these parameters remains challenging^14^. The majority of successful optimization efforts exploit the binding of biotinylated metal cofactors to (strept)avidin^10^; therefore, the development of new scaffolds capable of imparting high levels of selectivity metal catalysts could significantly expand the scope of ArM catalysis^9^. Furthermore, the ArM-catalysed reactions explored to date rarely involve catalytic intermediates that can react irreversibly with water in a deleterious maner, and no examples have been reported in which an ArM can mitigate this undesired reactivity^7^.

We recently developed a new method for ArM formation via strain-promoted azide–alkyne cycloaddition (SPAAC) of bicyclo[6.1.0]nonyne (BCN)-substituted cofactors and scaffold proteins containing a genetically encoded L-4-azidophenylalanine (Z) residue (Fig. 1a)^15^. Unlike non-covalent methods for ArM formation, this approach allows the use of any desired protein as a scaffold, and, unlike most covalent methods, the bioorthogonality of SPAAC eliminates the need to remove residues (for example, cysteine) in the scaffold that might react with electrophiles used in conventional bioconjugation methods (for example, maleimides)^7^. ArM formation from various cofactors, including the Esp-based^16^ dirhodium cofactor **1** (Fig. 1b), was demonstrated with a range of protein scaffolds, but no selectivity was observed in reactions catalysed by these systems. We attributed this lack of selectivity to the inability of the protein scaffolds selected for bioconjugation method development to fully encapsulate the cofactors selected for catalysis. Given the broad range of reactions catalysed by dirhodium complexes (Fig. 1c), including cyclopropanation and X–H insertion (X=C, N, O, and so on)^17^, and the selectivity challenges that persist for many of these reactions^18^, we sought to identify a scaffold protein that could impart selectivity to **1**. This would validate our hypothesis regarding the poor selectivity of our initial ArM designs, illustrate the importance of scaffold selection in ArM design and provide a platform for the development of future ArMs using different metal cofactors.

Here we show that a prolyl oligopeptidase (POP) scaffold can be used to generate dirhodium ArMs that catalyse asymmetric cyclopropanation. Genetic optimization of these ArMs led to high levels of enantioselectivity and reduced levels of byproducts resulting from the reaction of catalytic intermediates with water.

## Results

### Scaffold selection and bioconjugation

**Figure 2:**
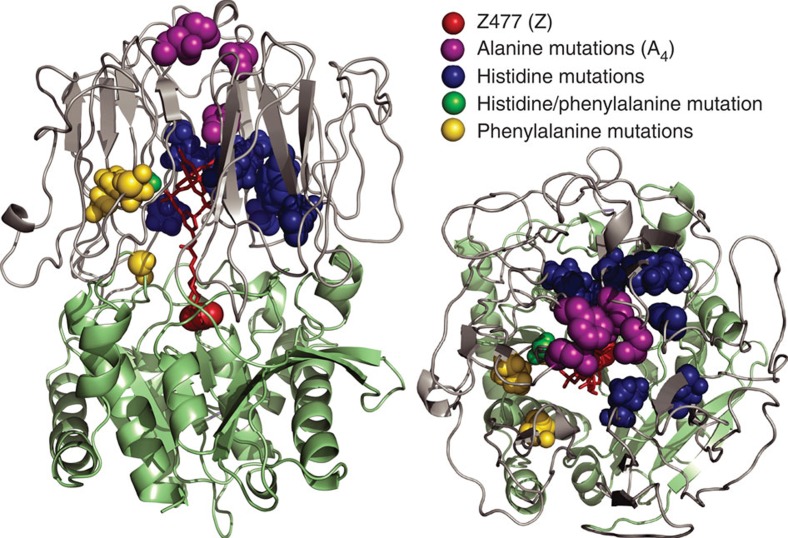
Homology model^21^ of *Pfu* POP. The hydrolase domain is shown in green, the propeller domain is shown in grey and cofactor **1** linked at Z477 is shown in red. Sites of different mutations introduced into *Pfu* POP are shown as coloured spheres.

An extensive search of different protein X-ray structures in the protein data bank (PDB) led to the identification of several members of the prolyl oligopeptidase family as potential ArM scaffolds because of their roughly cylindrical shapes (30 × 60 Å) and large internal volumes (5–8 × 10^3^ Å^3^) for cofactor enclosure^19^. This family includes POPs, dipeptidyl peptidases IV, oligopeptidases B and acylaminoacyl peptidases. All of these enzymes share a common fold comprising an α/β hydrolase domain, which contains a Ser-Asp-His triad for amide bond hydrolysis, capped by a β-barrel domain. We initially selected a POP from *Pyrococcus furiosus* (*Pfu*) as a scaffold for ArM formation because of its high thermal stability^20^. Despite the abundance of POP structures in the PDB, however, the structure of *Pfu* POP has not yet been solved; therefore, a previously reported homology model^21^ of this enzyme was used for initial engineering efforts (Fig. 2). An amber codon was introduced into the POP gene to replace the catalytically active serine (S477) with a Z residue (Z477), abolish the native proteolytic activity of the enzyme and position the cofactor centrally within the active site. A POP gene whose codon usage was optimized for expression in *E. coli* was used as a template for genetic manipulation, and the resulting scaffold, POP-Z, was expressed in high yield (>100 versus ∼10 mg l^−g^ before codon optimization) with essentially quantitative Z incorporation. Unfortunately, however, no reaction occurred between POP-Z and **1**. POP variants in which other active site residues had been replaced with Z proved similarly unreactive towards **1**, but rapid reaction of surface-exposed Z residues was observed^22^.

POP family enzymes have been crystallized in open and closed conformations^23^,^24^ and are proposed to sample both conformations during catalysis^25^. Active site residues, including Z477, should be accessible for bioconjugation in the open conformation. We hypothesized that the lack of POP-Z bioconjugation resulted from the enzyme existing predominantly in the closed conformation under the reaction conditions explored^26^ and that **1** is too large to enter the POP-active site in this conformation. Because the closed conformation of POP possesses the cylindrical shape and solvent-sequestered active site that we hoped to exploit for ArM catalysis, this indicated that POP-Z modification would be required for bioconjugation.

Early proposals for the substrate specificity of POP, which acts only on short peptides (<30 residues), invoked the entry of these substrates through a small pore at the end of the β-barrel domain where the β-sheets comprising this domain converge^27^. More recent studies have concluded that substrates do not enter via this pore and that it does not appear to be relevant to POP protease activity^24^,^28^; however, we envisioned that this pore could be coopted for ArM formation^29^. Examining the pore structure of *Pfu* POP in the aforementioned homology model^21^ suggested that four residues (E104, F146, K199 and D202) were largely responsible for blocking access to the active site (Fig. 2). We mutated these residues in POP-Z to alanine, and the resulting protein, POP-ZA_4_, underwent rapid bioconjugation in the presence of cofactor **1** at 4 °C to form POP-ZA_4_-**1**. The simplest explanation for this result is that the A_4_ mutations expand the pore to enable cofactor access to the POP-active site. It may also be that these mutations facilitate conformational changes that enable domain opening^24^, and subsequent experiments will be required to differentiate these mechanisms. Regardless of the mechanism by which the A_4_ mutations enable bioconjugation of POP-Z, the success of this strategy highlights the potential for mutagenesis to allow the use of otherwise unreactive proteins as ArM scaffolds.

### ArM catalysis and optimization

Owing to variations in the extent of bioconjugation observed for different POP variants^15^, ArM concentration was determined by multiplying the total protein concentration in purified ArM/scaffold mixtures by the ratio of the high-resolution electrospray ionization-mass spectrometry (ESI-MS) peak intensities of the ArM and scaffold in these mixtures (Supplementary Fig. 1 and Supplementary Table 2). In this way, consistent dirhodium loadings were used regardless of the extent of bioconjugation (Supplementary Table 3). Following this procedure, the catalytic activity of 1 mol% POP-ZA_4_-**1** was examined using the cyclopropanation of styrene with donor–acceptor diazo **2** as a model reaction^30^, and cyclopropane **3** was formed as a single diastereomer in 19% yield and 11% ee (Table 1, Entry 1). This enantioselectivity, while low, showed that the POP scaffold could impart selectivity to cofactor **1** (ref. 16), unlike previously described scaffolds^15^. A number of additional linkage sites for **1** within the POP-active site were examined; however, none provided significantly higher selectivity than Z477. A range of reaction parameters (buffer, co-solvent, pH, ad so on) were also explored, and it was observed that high concentrations of NaCl and NaBr provided a marked increase in enantioselectivity (up to 38% ee, Table 1, Entry 3). Using PIPES buffer in place of Tris also provided a slight increase in enantioselectivity for some substrates. While salt and medium effects on POP-catalysed peptide hydrolysis have been reported^21^, the mechanism by which they have an impact on POP-ZA_4_-**1** catalysed cyclopropanation is not clear, and further studies will be required to rationalize these improvements.

Several researchers have hypothesized that cofactor movement within protein scaffolds can reduce ArM selectivity^7^, and various strategies intended to decrease cofactor movement, including two-point covalent attachment^31^ and metal coordination by histidine^32^, have been shown to increase ArM selectivity. We pursued the latter strategy to improve POP-ZA_4_-**1**, given the established success of this method in peptide-based dirhodium catalysts^33^. Histidine mutations were individually introduced at several residues within POP-ZA_4_ that projected towards the POP-active site cavity (99, 139, 141, 197, 209, 218, 219, 251, 283 and 328, see Fig. 2), and the enantioselectivity of the resulting ArMs was examined. Of these, L328H provided a significant increase in both conversion and enantioselectivity in the corresponding ArM, POP-ZA_4_-H-**1** (Table 1, Entry 8). We hypothesize that histidine coordination to the proximal Rh of **1** projects the distal Rh towards a specific region of the POP-active site and that the improved enantioselectivity of POP-ZA_4_-H-**1** results from the ability of residues near the distal rhodium atom to impart selectivity to cyclopropanation reactions occurring at this centre. With the aim of further improving the selectivity of this ArM, we mutated to phenylalanine several residues (64, 97, 99 and 594, see Fig. 2) near and projecting towards the putative location of the distal Rh (Table 1, Entries 9–14). While only F99 improved enantioselectivity, the F99/F97 and F99/F594 double mutants provided modest further improvements, ultimately leading to cyclopropanation with 92% ee using POP-ZA_4_-HFF-**1** (Table 1, Entry 14, Supplementary Fig. 2).

### ArM selectivity and specificity

Several aspects of the activities exhibited by ArMs in the POP-ZA_4_-HFF-**1** lineage deserve comment. First, the majority of selective ArMs developed to date involve either chiral-at-metal complexes or complexes with flexible ligands, and Ward has proposed that a relay of chirality from a protein scaffold (streptavidin) to these complexes could contribute to the enantioselectivity of these systems^10^. This mechanism of asymmetric induction seems unlikely for POP-ZA_4_-HFF-**1**, given the rigidity of **1**. The observed selectivity is more consistent with direct interactions between active site residues, substrates and catalytic intermediates^9^,^34^, which suggests that the POP scaffold could be used to impart selectivity to a wide range of metal complexes. It is also interesting to note that high selectivity is achieved despite the C_s_ symmetry of the BCN moiety in **1**, which would lead to enantiomeric cycloadducts on reaction with the Z residue in POP-ZA_4_-HFF-**1** (Supplementary Fig. 8). The extended conformation of the exo BCN diastereomer and the distance between the BCN moiety and the dirhodium centre in **1** could render structural differences between these enantiomers small enough that they have only a minor impact on cofactor position in the POP active site. On the other hand, POP could impart enantioselectivity to the stoichiometric cycloaddition, in which case mutations introduced to improve the enantioselectivity of ArM catalysed cyclopropanation could have done so by improving cycloaddition enantioselectivity and thus ArM diastereopurity (Supplementary Fig. 8). Structural studies of the POP ArMs described in this work are underway and could shed light on these possiblities.

**Figure 3:**
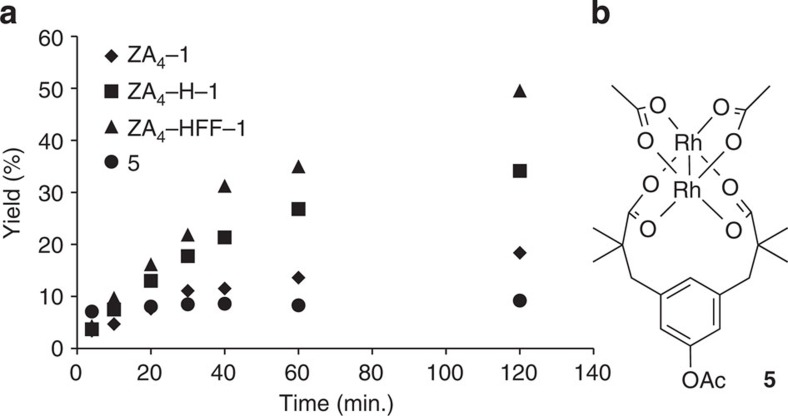
Kinetic analysis of cyclopanation reactions. (**a**) Comparison of product yield versus time for cyclopropantion of styrene using **2** catalysed by various ArMs or **5** (0.5 mol%). (see Supplementary Fig. 3) (**b**) Structure of **5**.

Second, the only screening criterion used in our engineering effort was improved enantioselectivity, but increased conversion was also observed (Table 1). This trend is particularly notable relative to soluble small molecule catalyst **5**, which gave lower conversion than any of the ArMs investigated (Table 1, Entry 15, Fig. 3b). While detailed kinetic analysis of these reactions was complicated by poor substrate solubility at elevated concentrations, monitoring the reaction of styrene with diazo **2** shows that ArMs that provide improved enantioselectivity also have increased cyclopropanation rates (Fig. 3). The rate of diazo consumption by these ArMs is well below that of **5**, which provides nearly instantaneous consumption of **2** (Supplementary Figs 4 and 5). Subsequent additions of **2** to reactions catalysed by **5** lead to similarly rapid conversion of this species with only minor increases in cyclopropanation conversion, indicating that **5** remains active even after the first aliquot of **2** is consumed (Supplementary Fig. 6). The discrepancy between diazo consumption and cyclopropanation catalysed by **5** results from the poor substrate specificity of this catalyst in aqueous solution. Under these conditions, formal carbene insertion into the O–H bond of water (rather than the olefin π bond) readily occurs to form α-hydroxyester **4** (ref. 17) a problem that has long-complicated aqueous dirhodium-catalysed carbene insertion reactions^18^. Importantly, however, the cyclopropane/α-hydroxyester ratio (**3**/**4**) increases from 0.4 using **5** to 2.4 using POP-ZA_4_-HFF-**1**. This sixfold increase occurred in increments that parallel increases in enantioselectivity (Table 1, Entries 4, 8, 11, 14). Together, these conversion, rate and selectivity data highlight the improved complementarity between POP and styrene in the engineered ArMs. The specificity of POP-ZA_4_-HFF-**1** for styrene over water ultimately leads to increased cyclopropanation conversion even though **5** provides much faster conversion of **2** under the reaction conditions (Supplementary Figs 4 and 5).

Third, despite the specificity of POP-ZA_4_-HFF-**1** for styrene over water, this ArM also catalyses enantioselective cyclopropanation of different styrenes using a variety of donor–acceptor diazo compounds (Table 2). Electron-withdrawing and electron-donating substituents were tolerated on the aromatic groups of both the styrene and diazo substrates. A styrenyl diazo substrate also reacted, albeit with significantly reduced selectivity relative to aryl diazoacetates. Ethyl diazoacetate, an acceptor-only carbene precursor, was also a competent substrate, but provided negligible enantioselectivity.

**Figure 4:**
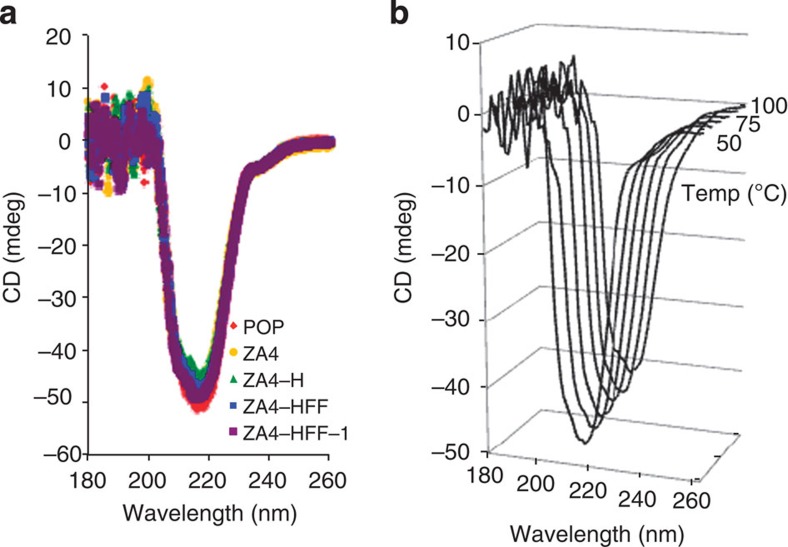
CD spectra for POP variants and ArMs. (**a**) Comparing different constructs (10 μM). (**b**) CD spectra of POP-ZA_4_-HFF acquired at 10 °C intervals from 50 to 100 °C (see also Supplementary Fig. 7).

The unique catalytic properties of POP-ZA_4_-HFF-**1** result from the introduction of eight mutations and dirhodium cofactor **1** into the interior of the POP scaffold, far more mutations than described in most ArM efforts^7^. Despite these perturbations, essentially identical circular dichroism (CD) spectra were obtained for several POP variants and POP-ZA_4_-HFF-**1**, suggesting little difference in secondary structures of these proteins (Fig. 4a)^35^. Remarkably, the CD spectrum of POP-ZA_4_-HFF-**1** remains unchanged up to 100 °C (Fig. 4b), indicating that the stability of POP itself is also not reduced to a relevant extent. This stability clearly highlights the utility of protein scaffolds from hyperthermophilic organisms that can form robust ArMs even when extensive mutagenesis is required to achieve high selectivity and will greatly facilitate further efforts to evolve ArMs derived from the POP scaffold^36^. Of the mutations introduced, L328H led to the largest improvements in both selectivity, conversion and activity (Table 1, entry 8). As previously noted, this mutation was introduced based on the improved selectivity of peptide-based dirhodium catalysts containing a histidine residue capable of coordinating to Rh^33^. It is important to note, however, that axial coordination of ligands to dirhodium complexes in peptide and small molecule catalysts typically leads to decreased activity^33^. Given the difference in the effects of histidine incorporation into peptide catalysts and POP-ZA_4_-HFF-**1**, several additional ArM variants were examined to clarify the role of H328 in POP-ZA_4_-HFF-**1** (Table 1, entries 4–8). First, POP-ZA_4_-L328F-**1** was prepared to examine the impact of a non-coordinating aromatic residue at position 328. The L328F variant possesses significantly lower selectivity than the L328H variant, suggesting that purely steric factors are not responsible for the improved selectivity of the latter. In addition, the L328M and L328C variants show that other residues capable of coordinating to Rh also improve ArM selectivity. The structural differences between histidine, methionine and cysteine suggest that their common metal-coordinating ability is responsible for the improved selectivity ArMs containing these residues, including POP-ZA_4_-HFF-**1**. Initial attempts to characterize histidine coordination to **1** in this ArM via NMR spectroscopy^37^,^38^ and ultraviolet–vis spectroscopy^33^,^39^,^40^ have been complicated by the high molecular weight of POP (*ca.* 70 kDa) and the weak absorbance associated with the diagnostic Rh–Rh π*–σ* transition^41^ in **1**, respectively. Further spectroscopic and crystallographic analysis of this ArM is underway to rigorously characterize the nature of cofactor binding within its active site and thus provide a mechanistic rationale for its high selectivity and improved specificity.

## Discussion

The unique structure of *Pfu* POP has allowed us to engineer ArMs using this enzyme and **1** to catalyse enantioselective cyclopropanation. This effort required genetic incorporation of a Z residue to covalently link **1**, four alanine mutations (A_4_) to enable cofactor entry into the POP active site and three additional active site mutations (HFF) to improve the enatioselectivity and substrate specificity of the initial ArM construct. The use of SPAAC for cofactor bioconjugation provided the flexibility to choose POP as a scaffold because of its physical properties (shape, size and stability), rather than native cofactor-binding ability^10^,^42^, which, in turn, allowed the extensive mutagenesis required for ArM formation and selective catalysis. Despite this mutagenesis, the optimized ArM, POP-ZA_4_-HFF-**1**, is extremely stable (Fig. 4), which will facilitate subsequent evolution^36^ of ArMs with improved activity and selectivity for different substrates and reactions.

POP-ZA_4_-HFF-**1** accepts a range of styrene and donor–acceptor carbene precursor^30^ substrates. In the latter respect, it contrasts with recent reports from Arnold^43^,^44^ and Fasan^45^ who have shown that naturally occuring haeme proteins catalyse olefin cyclopropanation using ethyl diazoacetate (an acceptor-only carbene precursor). Furthermore, while exciting developments, these systems exploit the native folds of enzymes and proteins that evolved to bind haeme in a manner appropriate for interacting with substrates in well-defined active sites. In contrast, selective ArM catalysis involves incorporating a synthetic metal complex into a protein scaffold and engineering an active site suitable for imparting selectivity to that complex. In the current case, this effort led to improved specificity of POP-ZA_4_-HFF-**1** for styrene over water, which is remarkable, given the known reactivity of dirhodium donor–acceptor carbene intermediates^46^ towards water^18^ and suggests that similar control of other water-sensitive organometallics could be possible using the solvent-sequestered POP active site. This contrasts significantly with peptide scaffolds, which, while being capable of imparting high levels of selectivity to dirhodium catalysts, require the use of organic solvents or a large excess of diazo substrate^47^ to compensate for reactivity of carbene intermediates with water. Given the wide range of reactions catalysed by dirhodium complexes^17^ and the selectivity of POP-ZA_4_-HFF-**1**, we anticipate that dirhodium ArMs will provide many unique opportunities for selective catalysis. Furthermore, the ability of POP to impart selectivity to the rigid dirhodium complex suggests that similar selectivity should be possible for a wide range of additional metal complexes regardless of their stereochemical properties^10^. POP will thus serve as a robust scaffold to explore this possibility and to study the effects of attractive interactions, molecular recognition and scaffold dynamics on transition metal catalysis.

## Methods

### Materials

Unless otherwise noted, all reagents were obtained from commercial suppliers and used without further purification. Solvent for organic synthesis, including benzene, dimethylformamide, acetonitrile, pentane, tetrahydrofuran (THF) and methylene chloride were obtained from a PureSolv MD solvent purification system by Innovative Technology (solvent deoxygenated by N_2_ sparge and dried over alumina). Acetonitrile for other applications was purchased from Fisher Chemical, HPLC grade. Deuterated solvents were obtained from Cambridge Isotope laboratories. Silicycle silica gel plates (250 mm, 60 F254) were used for analytical thin-layer chromatography, and preparative chromatography was performed using SiliCycle SiliaFlash silica gel (230–400 mesh). Rh_2_(R-DOSP)_4_ was purchased from Strem Chemicals. Azide Agarose was purchased from Click Chemistry Tools LLC. Labquake Tube Shaker/Rotators were purchased from Thermo Scientific (Catalogue (Cat)# 4002110Q). Cofactor **1** (ref. 15) and aryldiazo acetates^48^,^49^,^50^ were prepared using literature methods.

Plasmid pEVOL-pAzF was provided by the Schultz group of the Scripps Research Institute (La Jolla, CA)^51^. A codon-optimized gene for POP^20^ was obtained from GenScript USA Inc. (Piscataway, NJ) and cloned into the NcoI and XhoI restriction sites of the pET28a plasmid vector. *E. coli* DH5α and BL21 (DE3) cells were purchased from Invitrogen (Carlsbad, CA). NcoI and XhoI restriction enzymes, T4 DNA ligase, Taq DNA polymerase and Phusion HF polymerase (Cat# 530S) were purchased from New England Biolabs (Ipswitch, MA). Luria broth (LB), rich medium (2YT) and agar media were purchased from Research Products International (Mt. Prospect, IL). Gel extraction (Cat# 28706) and plasmid isolation (Cat# 27106) kits were purchased from QIAGEN Inc. (Valencia, CA) and used according to the manufacturer's instructions. A DNA purification kit (Zymo, Cat# D4004) was purchased from Zymo research (Irvine, CA) and used as recommended for purifying DNA following PCRs. All genes were confirmed by sequencing at the University of Chicago Comprehensive Cancer Center DNA Sequencing & Genotyping Facility (900 E. 57th Street, Room 1230H, Chicago, IL 60637). Electroporation was carried out on a Bio-Rad MicroPulser using method Ec2. Ni-nitrilotriacetic acid (Ni-NTA) resin and Pierce BCA Protein Assay Kits (Cat# 23225) were purchased from Fisher Scientific International Inc. (Hampton, NH), and the manufacturer's instructions were followed when using both products (for Ni-NTA resin, 8 ml resin was used with buffers delivered by a peristaltic pump at a rate of 1 ml min^−1^, in a 4 °C cold cabinet). Amicon 30-kD spin filters for centrifugal concentration were purchased from EMD Millipore (Billerica, MA) and used at 4,000*g* at 4 °C.

### General procedures

Reactions for cofactor and substrate preparation were prepared in flame or oven-dried glassware under an inert N_2_ atmosphere using either syringe or cannula techniques. Thin-layer chromatography plates were visualized using 254-nm ultraviolet light. Flash column chromatography was carried out using Silicycle 230–400 mesh silica gel. ^1^H and ^13^C NMR spectra were recorded at 500 and 126 MHz, respectively, on a Bruker DMX-500 or DRX-500 spectrometer, and chemical shifts are reported relative to residual solvent peaks. Chemical shifts are reported in p.p.m. and coupling constants are reported in Hz. Yields for ArM-catalysed reactions were determined by HPLC with 1,2,4-trimethoxybenzene as the internal standard and reported as the average of two trials from the same batch of ArM set up in parallel. High-resolution ESI mass spectra were obtained using an Agilent Technologies 6,224 time of flight (TOF) LC/MS. Low-resolution ESI mass spectra were obtained using Agilent 6,130 LC-MS. Amicon 50-ml 30-kD cutoff centrifugal filter was used to concentrate or wash protein solutions. Protein concentrations were measured using the Pierce BCA Protein Assay Kit and protein stocks were then stored at −80 °C until use. CD spectra were obtained on a JASCO J-1,500 CD Spectrometer.

### Scaffold cloning and mutagenesis

Alanine mutations (at positions E104A, F146A, K199A and D202A), histidine mutations (at positions G99H, P139H, I141H, I197H, T209H, E218H, V219H, Y251H, E283H and L328H) and phenylalanine mutations (at positions S64F, L97F, G99F and G594F) were introduced into the codon-optimized *Pfu* POP gene by site-directed overlap extension PCR^52^. To introduce mutations, two separate PCRs were performed, each using a perfectly complementary flanking primer at the 5′ and 3′ ends of the sequence and a mutagenic primer (Supplementary Table 1). The PCR conditions were as follows: Phusion HF buffer 1 × , 0.2 mM dNTPs each, 0.5 μM forward primer, 0.5 μM reverse primer, 0.02 U μl^−1^. Phusion polymerase and 0.5 ng ml^−1^ template plasmid. The PCR thermocycler programme was as follows: 98 °C, 60 s; 95 °C, 20 s; 54 °C, 45 s; 72 °C, 120 s; 72 °C, 10 min; repeat steps 2–4 25 × .

The resulting two overlapping fragments that contained the base pair substitution were then assembled in a second PCR using the flanking primers resulting in the full-length mutated gene. The same PCR programme was used with a slightly altered annealing temperature (step 3) of 52 °C. Nucleotide sequences for the all the primers are summarized in Supplementary Table 1. PCR-amplified fragments and plasmid vector pET28a were restriction digested with NcoI and XhoI enzymes in recommended buffer at 37 °C for 2 h. Digested DNA was purified using agarose gel (1% agarose) electrophoresis and isolated using a Qiagen DNA extraction kit. Ligation reactions were conducted using a molar ratio of 1:3 (plasmid:insert) in 10 μl reaction mix. A typical ligation reaction contained 3 ng ml^−1^ digested plasmid vector, 9 ng ml^−1^ of the insert, 1 μl 10 × ligase buffer and 1 U ml^−1^ ligase. The reaction mixture was incubated at 16 °C overnight, purified using a Zymo DNA purification kit and transformed into *E. coli* DH5 cells. Cells were spread on LB kanamycin plates (6.25 g LB powder mix, 4 g agar, 250 ml DDI water, 0.05 mg ml^−1^ kanamycin) before recovering in SOC medium for 1 h at 37 °C. Plates were incubated at 37 °C overnight; individual colonies that appeared next day were tested for gene fragments using colony PCR. Clones that showed amplification for desired fragments were inoculated on LB broth containing 0.05 mg ml^−1^ kanamycin and grown overnight at 37 °C, 250 r.p.m. Recombinant plasmid from these overnight grown cultures were isolated using a Qiagen gel extraction kit and sequenced using T7 forward and reverse primers.

### Scaffold expression

Electrocompetent *E. coli* BL21 (DE3) cells were co-transformed with pET28a-POPZA_4_ and pEVOL-pAzF^51^. Transformants were allowed to recover in SOC medium (37 °C, 50 min), the mixture was spread on LB kanamycin+chloramphenicol agar plates (6.25 g LB powder mix, 4 g agar, 250 ml DDI water, 0.05 mg ml^−1^ kanamycin and 0.05 mg ml^−1^ chloramphenicol), and the plates were incubated at 37 °C for 16 h. Several colonies appeared on overnight-incubated plates; a single colony from this plate was inoculated in 5 ml 2YT medium having antibiotics with the same concentrations as above. The culture was incubated overnight at 37 °C with constant shaking at 250 r.p.m. On the following day, 5 ml of the overnight cultures was used to inoculate 500 ml of fresh 2YT media having the same antibiotics in 5 l Erlenmeyer flask. The culture was incubated at 37 °C, 250 r.p.m., and protein expression was induced by adding 1 mM isopropylthiogalactoside, 2 mM 4-Azido-phenyl alanine and 1% (w/v) L-arabinose when OD_600_ reached 1. The induced culture was allowed to grow for 12 h, and then the cells were harvested by centrifugation at 4 °C, 3,000*g* for 20 min. Cell pellets were re-suspended in 30 ml PBS (pH 7.5) and sonicated (40 amplitude, 30 s burst, 10 min total process). Lysed culture was clarified by centrifugation at 16,000*g*, 4 °C for 30 min and supernatant thus obtained was purified by Ni-NTA resin using the manufacturer's instructions. Purified protein was buffer-exchanged to 10 mM Tris (pH 7.5) and measured using Pierce BCA Protein Assay Kit as recommended.

### Bioconjugation

A solution of the POP-Z mutant (480 μl, 75 μM in 50 mM Tris-HCl buffer, pH 7.4) and a solution of cofactor **1** (120 μl, 0.75 mM in acetonitrile, 0.655 mg ml^−1^) were added to a 1.5-ml microcentrifuge tube and shaken at 750 r.p.m. at 4 °C overnight. The final concentrations were: 60 μM POP-Z, 150 μM **1**, 20 vol% acetonitrile/Tris buffer. The resulting solution was treated with 100 μl azide agarose resin and rotated on a Labquake Tube Shaker/Rotator in a 4 °C cold cabinet for 24 h to remove excess cofactor. The suspension was then centrifuged at 5,000 r.p.m. for 3 min and the supernatant was transferred to a new microcentrifuge tube. The resin was rinsed twice with 600 μl 50 mM Tris-HCl buffer and centrifuged at 5,000 r.p.m. for 3 min. These supernatants were combined with the first supernatant and buffer-exchanged to proper buffers for use in biocatalysis or characterization (Supplementary Table 3).

### Biocatalysis

A solution of aryldiazoacetate (25 μl, 96 mM, in THF), styrene (25 μl, 485 mM, in THF) and POP-ZA_4_-X-**1** solution (500 μl, 48 μM) were added to a 1.5-ml microcentrifuge tube. The final concentrations of the reagents were as follows: 22 mM olefin, 4.4 mM aryldiazoacetate, 44 μM POP-ZA_4_-X-**1**. The resulting mixture was left shaking at 750 r.p.m. at 4 °C overnight. The reaction was quenched by adding 20 μl 1,2,4-trimethoxybenzene solution (30 mM, in THF) and 600 μl ethyl acetate. The mixture was vortexed and centrifuged (15,000*g*, 3 min). The top organic layer was collected and the bottom aqueous layer was extracted with 600 μl ethyl acetate twice. The organic layers were combined, evaporated and re-dissolved in 200 μl THF. THF solution (4 μl) of the crude product was analysed on RP-HPLC to determine conversions; 50 μl THF solution of the crude product was purified on preparative HPLC to isolate the cyclopropane product, which was analysed on NP-HPLC to determine enantioselectivities (Supplementary Tables 4–7).

## Additional information

**How to cite this article:** Srivastava, P. *et al*. Engineering a dirhodium artificial metalloenzyme for selective olefin cyclopropanation. *Nat. Commun.* 6:7789 doi: 10.1038/ncomms8789 (2015).

## Supplementary Material

Supplementary Information Supplementary Figures 1-8, Supplementary Tables 1-7, Supplementary Methods and Supplementary References

## Figures and Tables

**Figure 1:**
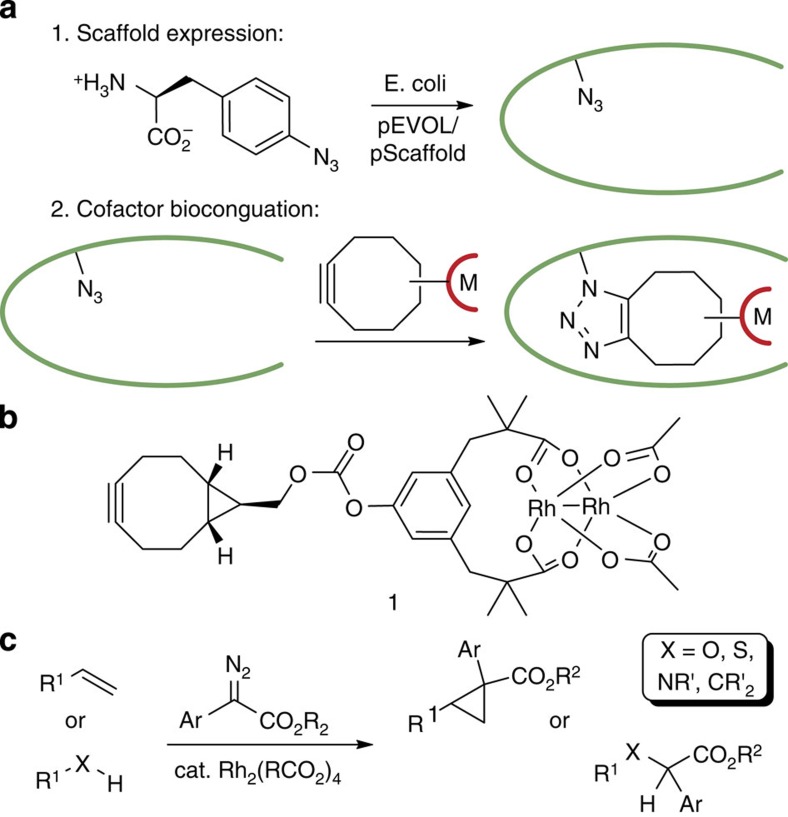
ArM formation and reactivity. (**a**) ArM formation using the SPAAC reaction. (**b**) Structure of cofactor **1**. (**c**) Representative reactions catalysed by dirhodium complexes.

**Table 1:** Optimization of reaction conditions and active site mutations.

					

**Table 2:** Representative substrate scope of POP-ZA_4_-HFF-**1**-catalysed cyclopropanation.

					
