# Supplementary material for: Engineering a dirhodium artificial metalloenzyme for selective olefin cyclopropanation
Source: Nat Commun. 2015 Jul 24;6:7789. doi: 10.1038/ncomms8789 (PMC4525152; doi:10.1038/ncomms8789)
Supplement: Supplementary Information — Supplementary Figures 1-8, Supplementary Tables 1-7, Supplementary Methods and Supplementary References [file ncomms8789-s1.pdf]

Supplementary Figures

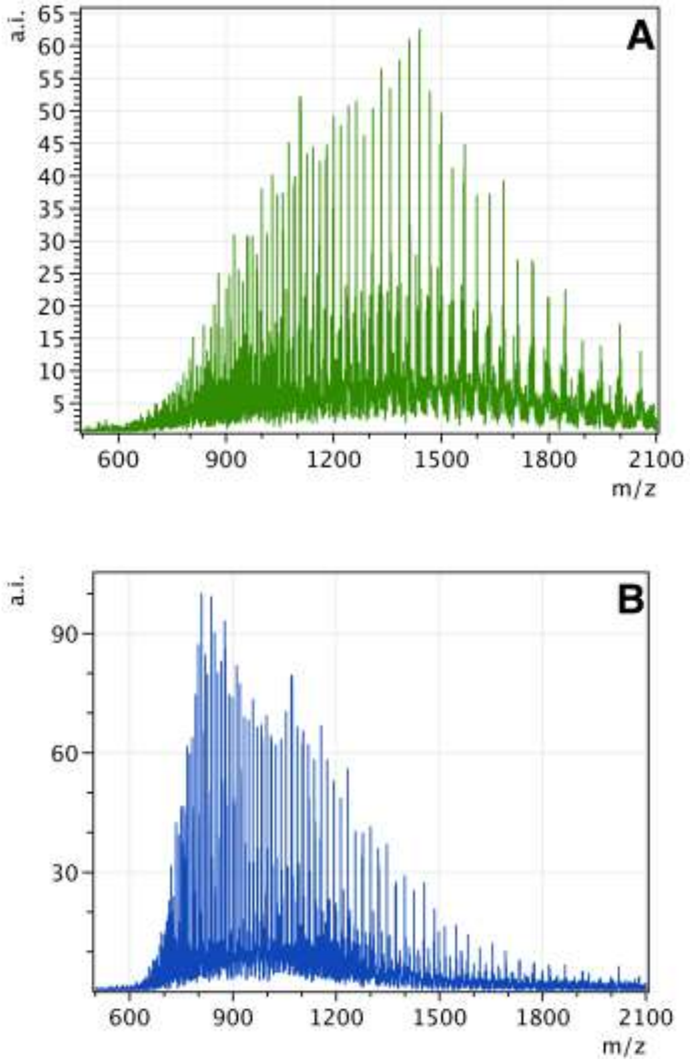

Supplementary Figure 1. Raw ESI spectra of **A)** POP- ZA_4-HFF (green) and **B)** POP-ZA_4-HFF-**1** (blue)

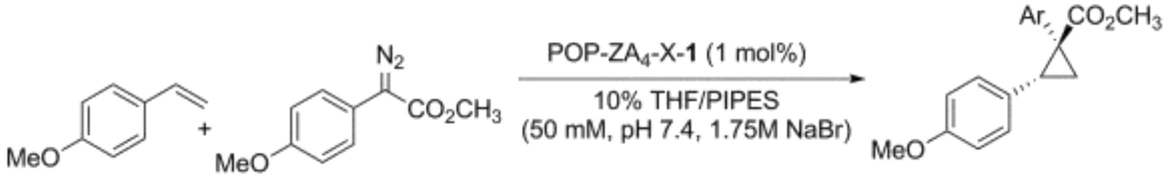

a)

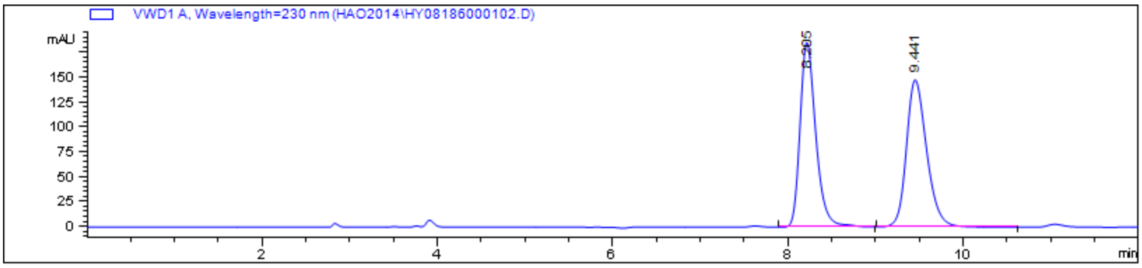

b)

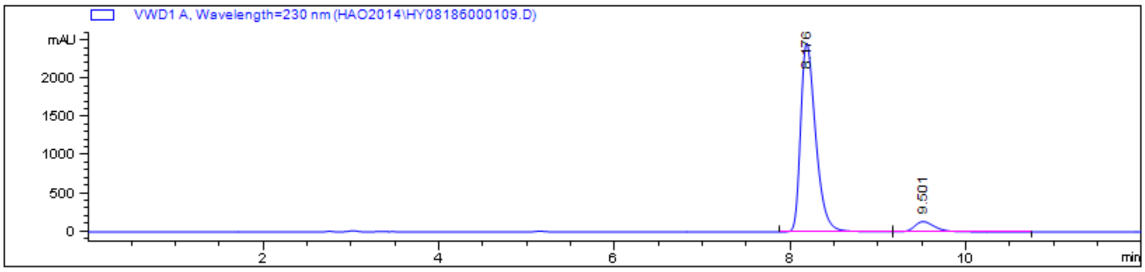

Supplementary Figure 2. Representative HPLC traces for a) a racemic mixture and b) enantiomeric mixture made by POP- ZA_4-HFF-**1**

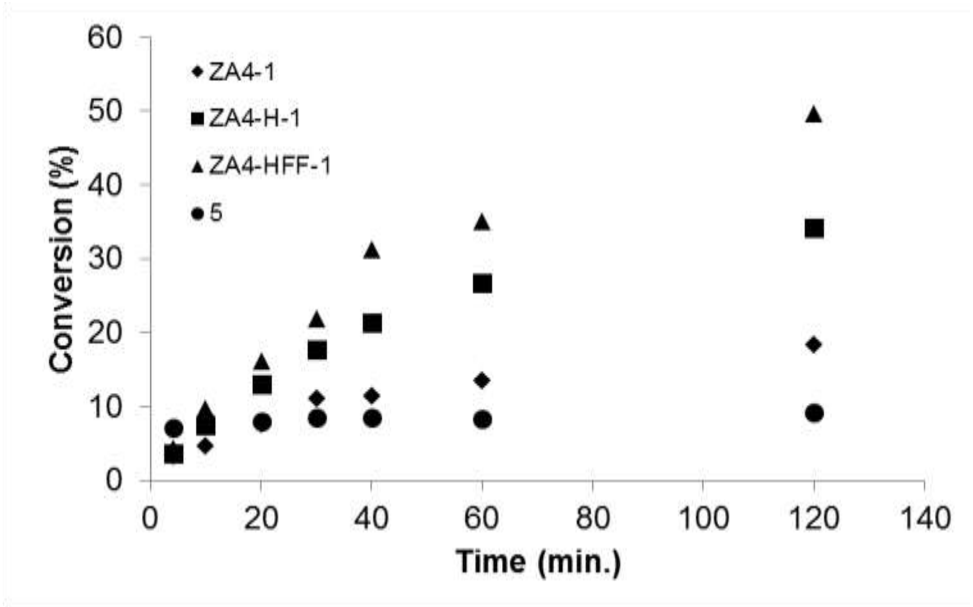

Supplementary Figure 3. Plot of percent yield for biocatalysis catalyzed by **5** and selective ArM hybrids as a function of time.

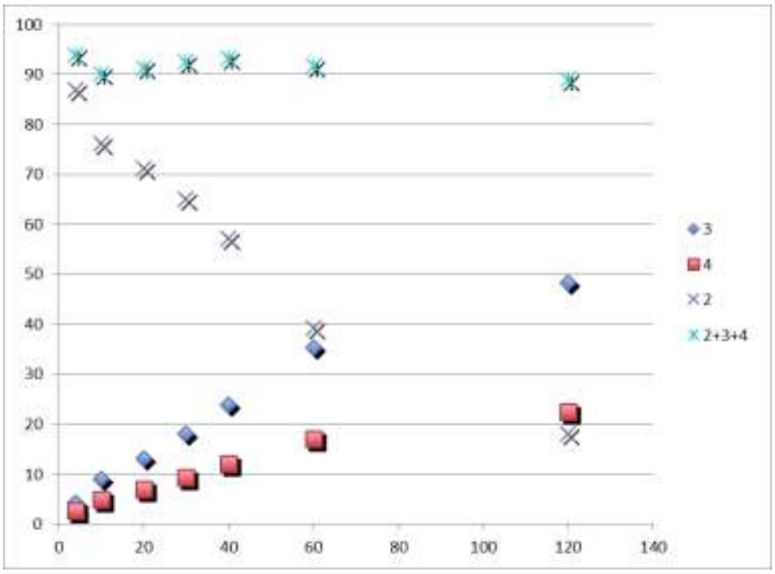

Supplementary Figure 4. Conversion of **2**, **3**, **4** over time for POP- ZA_4-HFF-**1**.

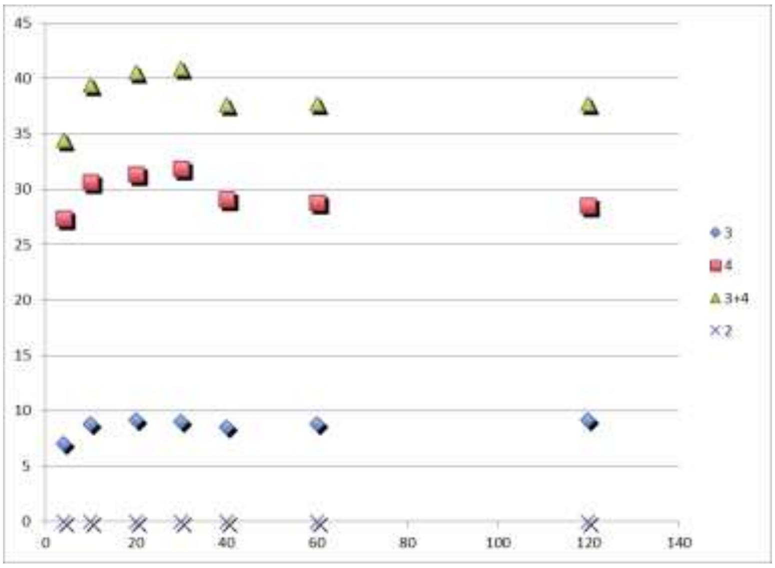

Supplementary Figure 5. Conversion of **2**, **3**, **4** over time for **5**.

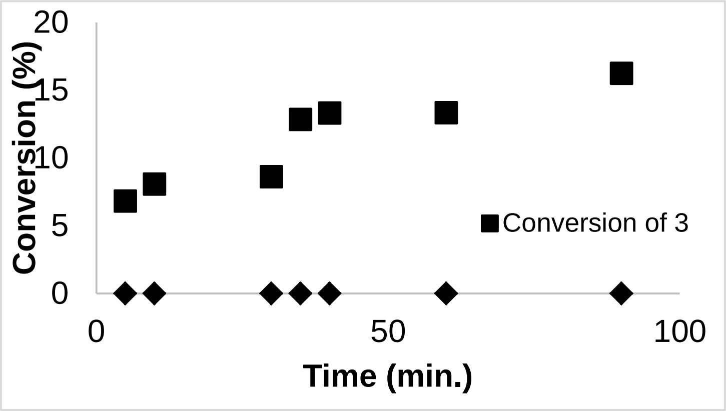

Supplementary Figure 6. Plot of conversion of **2** and **3** catalyzed by **5** (equal amount of **2** was added at 0, 30, 60 min). Following each addition, **2** is immediately consumed and additional conversion to **3** is observed.

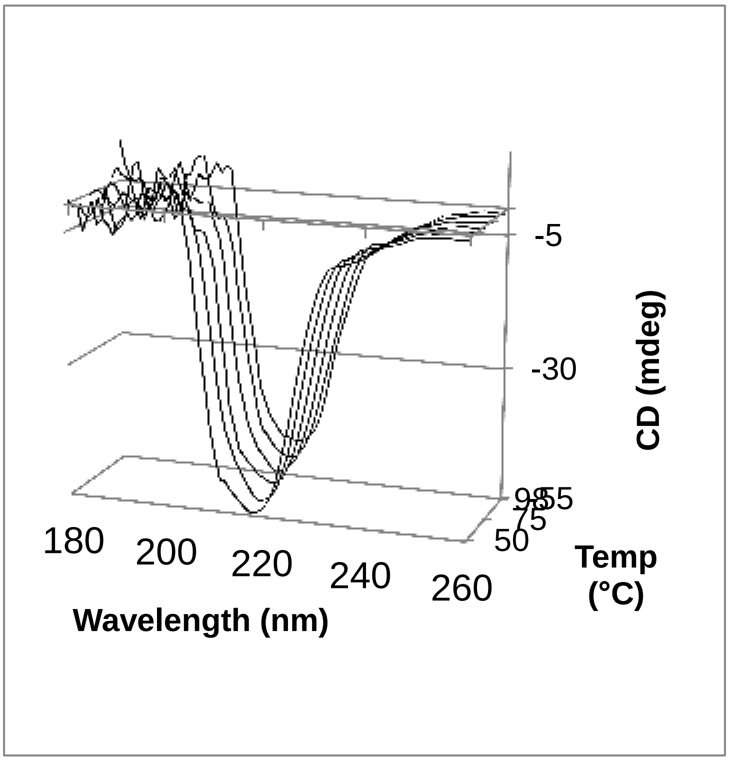

Supplementary Figure 7. CD temperature stability profile for WT POP

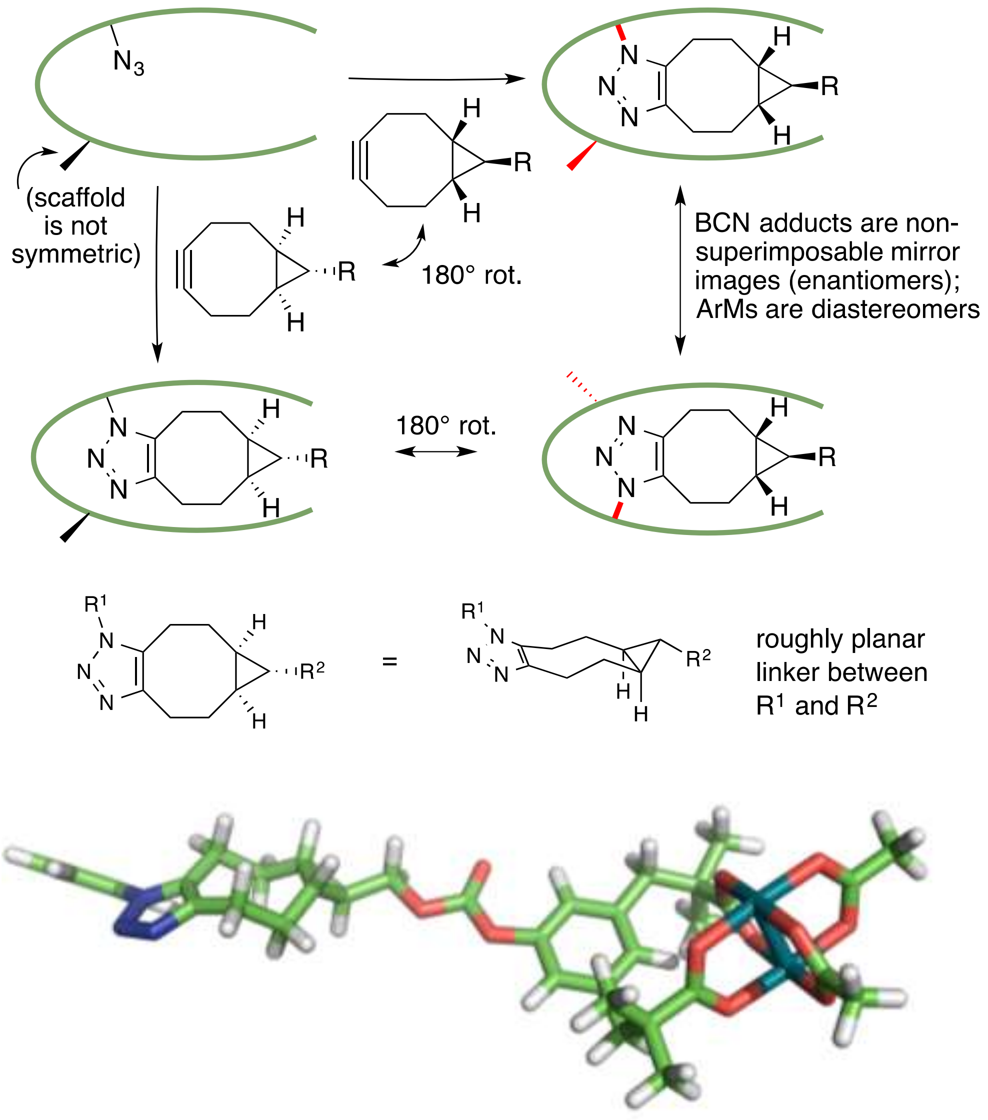

Supplementary Figure 8. Explanation of bioconjugation stereochemistry and a geometry optimized (DFT, B3LYP, LANL2DZ) structure of a phenylazide-**1** adduct.

Supplementary Tables

#	Primer name	Primer sequence
1	T7 for	5’-GCG AAA TTA ATA CGA CTC ACT ATA-3’
2	T7 rev	5’-TTA TGC TAG TTA TTG CTC AGC GG-3’
3	E104A for	5’- ACC ACG GAC GCG GAA GGT GAA A -3’
4	E104A rev	5’- T TTC ACC TTC CGC GTC CGT GGT -3’
5	F146A for	5’- AAC ATC ACC GCC CTG AAA GAT G -3’
6	F146A rev	5’- C ATC TTT CAG GGC GGT GAT GTT-3’
7	K199D202A for	5’- G TCC ATT CGC GCA AGC TCT GCT GGT AAA TTC G-3’
8	K199D202A rev	5’- C GAA TTT ACC AGC AGA GCT TGC GCG AAT GGA C-3’
9	POPz477 for	5’-A GCT TGG GGT CGT TAG AAT GGC GGT CTG-3’
10	POPz477 rev	5’-CAG ACC GCC ATT CTA ACG ACC CCA AGC T- 3’
11	HisG99 for	5’- TC CTG CTG CAG CAC TTT ACC ACG G-3’
12	HisG99 rev	5’- C CGT GGT AAA GTG CTG CAG CAG GA-3’
13	HisP139 for	5’- GAA GAA ATC AAA CAC TCC ATT TGG AAC-3’
14	HisP139 rev	5’- GTT CCA AAT GGA GTG TTT GAT TTC TTC -3’
15	HisI141 for	5’- C AAA CCG TCC CAC TGG AAC ATC ACC -3’
16	HisI141 rev	5’- GGT GAT GTT CCA GTG GGA CGG TTT G -3’
17	HisI197 for	5’- AT TTC ATG TCC CAC CGC GCA AGC TC-3’
18	HisI197 rev	5’- GA GCT TGC GCG GTG GGA CAT GAA AT-3’
19	HisT209 for	5’- TTC GCA ATC GTT CAC CTG ACG TAT GGT -3’
20	HisT209 rev	5’- ACC ATA CGT CAG GTG AAC GAT TGC GAA - 3’
21	HisE218 for	5’- AAC CAG GGC CAC GTC TAC ATT GG -3’
22	HisE218 rev	5’- CC AAT GTA GAC GTG GCC CTG GTT -3’
23	HisV219 for	5’- CAG GGC GAA CAC TAC ATT GGT CC -3’
24	HisV219 rev	5’- GG ACC AAT GTA GTG TTC GCC CTG -3’
25	HisY251 for	5’- GGC AAA CTG CAC ATC CTG ACC -3’
26	HisY251 rev	5’- GGT CAG GAT GTG CAG TTT GCC- 3’
27	HisE283 for	5’- AA TTT CCG CTG CAC TGG GCA GTC ATT GT -3’
28	HisE283 rev	5’- AC AAT GAC TGC CCA GTG CAG CGG AAA TT -3’
29	HisW284 for	5’- A TTT CCG CTG GAA CAC GCA GTC ATT GT -3’
30	HisW284 rev	5’- AC AAT GAC TGC GTG TTC CAG CGG AAA T -3’
31	HisL328 for	5’- CA CTG TAT CCG CAC GAT AAA GAC GA -3’
32	HisL328 rev	5’- TC GTC TTT ATC GTG CGG ATA CAG TG -3’
33	G99F for	5’- C CTG CTG CAG TTC TTT ACC ACG GA -3’

34	G99F rev	5’-TC CGT GGT AAA GAA CTG CAG CAG G- 3’
35	G594F for	5’-CA GGT CAC ATG TTC GCG TCG CCG G- 3’
36	G594F rev	5’- C CGG CGA CGC GAA CAT GTG ACC TG- 3’
37	L97F for	5’- T GAA GTC CTG TTT CAG GGC TTT ACC- 3’
38	L97F rev	5’- GGT AAA GCC CTG AAA CAG GAC TTC A- 3’
39	S64F for	5’- GGT ATT ATC GCT TTT TAT TCC GAA AAA- 3’
40	S64F rev	5’- TTT TTC GGA ATA AAA AGC GAT AAT ACC-3’

Supplementary Table 1. Nucleotide sequences for the primers.

	Calculated Mass (Da)	Observed Mass (Da)	Δ Mass (Da)
POP- ZA_4-HFF	71959.4	71935.6	-23.6
POP- ZA_4-HFF-1	72751.9	72745.3	-6.6

Supplementary Table 2. Calculated masses versus observed deconvoluted masses.

	Batch 1		Batch 2		Batch 3	
	Trial 1	Trial 2	Trial 1	Trial 2	Trial 1	Trial 2
Yield (%)	70	74	71	74	75	73
e.e.(%)	92	91	91	92	92	92

Supplementary Table 3. Summary of bioconversion (for the reaction in Supplementary Figure 2) catalyzed by duplicates from three independent batches of POP- ZA_4-HFF-**1**.

	Average yield (%)			
Time (min)	ZA_4-1	ZA_4-H328-1	ZA_4-HFF-1	**5**
4	3.4	3.7	4.3	7.1
10	4.7	7.5	9.7	8.2
20	7.6	13.0	16.2	8.0
30	11.1	17.7	21.9	8.5
40	11.5	21.3	31.3	8.6
60	13.6	26.8	35.0	8.3
120	18.4	34.1	49.6	9.2

Supplementary Table 4. Percent yield for biocatalysis catalyzed by **5** and selective ArM hybrids as a function of time

Time (min)	**2**%	**3**%	**4**%	(**2**+**3**+**4**)%
4	86.9	4.3	2.7	93.9
10	76.1	9.1	5.0	90.2
20	71.2	13.2	7.0	91.4
30	65.0	18.2	9.4	92.6
40	57.2	23.9	12.1	93.2
60	39.3	35.3	17.1	91.7
120	18.0	48.3	22.6	89.0

Supplementary Table 5. Conversion of **2**, **3**, **4** over time for POP- ZA_4-HFF-**1**.

Time (min)	**2**%	**3**%	**4**%	(**2**+**3**+**4**)%
4	0	7.0	27.4	34.4
10	0	8.8	30.6	39.4
20	0	9.2	31.4	40.5
30	0	9.0	31.9	40.9
40	0	8.5	29.1	37.7
60	0	8.8	28.8	37.7
120	0	9.2	28.5	37.7

Supplementary Table 6. Conversion of **2**, **3**, **4** over time for **5**.

Time (min)	**2**%	**3**%
5	0	6.8
10	0	8.0
30	0	8.6
35	0	12.8
40	0	13.3
60	0	13.3
90	0	16.2

Supplementary Table 7. Conversion of **2** and **3** catalyzed by **5** (equal amount of **2** added at 0, 30, 60 min).

Supplementary Methods

Materials

Unless otherwise noted, all reagents were obtained from commercial suppliers and used without further purification. Benzene, dimethylformamide (DMF), acetonitrile (ACN), pentane, tetrahydrofuran (THF), and methylene chloride (CH_2Cl_2) were obtained from a PureSolv MD solvent purification system by Innovative Technology (solvent deoxygenated by N_2 sparge and dried over alumina). Acetonitrile (ACN) was purchased from Fisher Chemical, HPLC grade. Deuterated solvents were obtained from Cambridge Isotope labs. Silicycle silica gel plates (250 mm, 60 F254) were used for analytical TLC, and preparative chromatography was performed using SiliCycle SiliaFlash silica gel (230-400 mesh). Rh_2(R-DOSP)$_4$ was purchased from Strem Chemicals. Azide Agarose was purchased from Click Chemistry Tools LLC. Labquake™ Tube Shaker/Rotators was purchased from Thermo Scientific (Catalog# 4002110Q). Cofactor **1** was prepared as previously described.[3] Plasmid pEVOL-pAzF was provided by the Schultz group of the Scripps Research Institute,

CA[1]. *E. coli* DH5α and BL21 (DE3) cells were purchased from Invitrogen (Carlsbad, CA). Nco I, Xho I restriction enzyme, T4 DNA ligase, Taq DNA polymerase and Phusion HF polymerase (Cat# 530S) were purchased from New England Biolabs (Ipswitch, MA). Luria broth (LB), rich medium (2YT) and Agar media were purchased from Research Products International (Mt. Prospect, IL). Qiagen DNA extraction kit (Cat# 28706) and plasmid isolation kit (Cat# 27106) were purchased from QIAGEN Inc. (Valencia, CA) and used according to the manufacturer's instructions. DNA purification kit (Zymo, Cat# D4004) was purchased from Zymo research (Irvine, CA) and used as recommended. All genes were confirmed by sequencing at the University of Chicago Comprehensive Cancer Center DNA Sequencing & Genotyping Facility (900 E. 57th Street, Room 1230H, Chicago, IL 60637). Electroporation was carried out on a Bio-Rad MicroPulser using method Ec2. Ni-nitrilotriacetic acid (Ni-NTA) resin and Pierce® BCA Protein Assay Kits (Cat# 23225) were purchased from Fisher Scientific International, Inc. (Hampton, NH), and the manufacturer's instructions were followed when using both products (for Ni-NTA resin, 8 mL resin was used with buffers delivered by a peristaltic pump at a rate of 1 mL/min, in a 4 °C cold cabinet). Amicon® 30 kD spin filters for centrifugal concentration were purchased from EMD Millipore (Billerica, MA) and used at 4,000 g at 4 °C.

General Methods

Unless otherwise specified, all reactions were prepared in flame or oven-dried glassware under an inert N_2 atmosphere using either syringe or cannula techniques. TLC plates were visualized using 254 nm ultraviolet light. Flash column chromatography was carried out using Silicycle 230-400 mesh silica gel. ^{1}H and ^{13}C NMR spectra were recorded at 500 MHz and 126 MHz, respectively, on a Bruker DMX-500 or DRX-500 spectrometer, and chemical

shifts are reported relative to residual solvent peaks. Chemical shifts are reported in ppm and coupling constants are reported in Hz. Yields were determined by HPLC with 1,2,4-trimethoxybenzene as the internal standard and reported as the average of two trials from the same batch of ArM set up in parallel. High resolution ESI mass spectra were obtained using an Agilent Technologies 6224 TOF LC/MS. Low resolution ESI mass spectra were obtained using Agilent 6130 LC-MS. Amicon® 50 mL 30 kD cutoff centrifugal filter was used to concentrate or wash protein solutions. Protein concentrations were measured using the Pierce® BCA Protein Assay Kit and protein stocks were then stored at -80 °C until use. Circular dichroism (CD) spectra were obtained on a JASCO J-1500 CD Spectrometer.

Standard cloning procedures and site directed mutagenesis:

A codon optimized gene for Prolyl oligopeptidase (POP) was obtained from GenScript USA Inc (Piscataway, NJ) and cloned into pET28a plasmid vector using NcoI and XhoI restriction sites. The gene was cloned upstream of a C-terminal hexa-histidine tag for Ni-NTA affinity chromatography. Alanine mutations (at positions E104A, F146A, K199A and D202A), histidine mutations (at positions G99H, P139H, I141H, I197H, T209H, E218H, V219H, Y251H, E283H, and L328H), and phenylalanine mutations (at positions S64F, L97F, G99F, G594F) were introduced into the POP gene by site directed overlap extension PCR[2]. To introduce mutations, two separate polymerase chain reactions were performed, each using a perfectly complementary flanking primer at the 5’ and 3’ end of the sequence and a mutagenic primer. The PCR conditions were as follows: Phusion HF buffer 1x, 0.2 mM

dNTPs each, 0.5 μM forward primer, 0.5 μM reverse primer, 0.02 U/μL Phusion polymerase and 0.5 ng/mL template plasmid.

Thermal cycler was programmed as:
1. 98 °C-60 seconds
2. 95 °C-20 seconds
3. 54 °C-45 seconds
4. 72 °C- 120 seconds
5. 72 °C-10 minutes
25 repeat cycles from #2 to #4

The resulting two overlapping fragments that contained the base pair substitution were then assembled in a second PCR using the flanking primers resulting in the full-length mutated gene. The same PCR program was used with a slightly altered annealing temperature of 52 °C. Nucleotide sequences for the all the primers are summarized in Supplementary Table 1.

PCR amplified fragments and plasmid vector pET28a were restriction digested with Nco I and Xho I enzymes in recommended buffer at 37 °C for 2 hours. Digested DNA was cleaned by agarose gel extraction using commercial kit before ligation. Ligation was set-up with a molar ratio of 1:3 (plasmid: insert) in 10 μL reaction mix. Typically a ligase reaction mix had 3 ng/mL digested plasmid vector, 9 ng/mL of the insert, 1 μL 10X ligase buffer and 1 U/mL ligase. The reaction mixture was incubated at 16 °C overnight, cleaned using DNA purification kits and transformed into *E. coli* DH5 cells. Cells were spread on LB kanamycin plates (6.25 g LB powder mix, 4 g agar, 250 mL DDI water, 0.05 mg/mL kanamycin) before recovering in SOC medium for 1 hour at 37 °C. Plates were incubated at 37 °C overnight; individual colonies that appeared next day were tested for gene fragments by colony PCR. Clones that showed amplification for desired fragments were inoculated on LB broth having

0.05 mg/mL kanamycin and grown overnight at 37 °C, 250 rpm. Recombinant plasmid from these overnight grown cultures were isolated using kit from Qiagen (Valencia, CA) and given for sequencing. Plasmid sequencing was done at the U Chicago sequencing facility and T7 for and T7 rev primers were used for sequencing reactions.

Standard expression and purification procedure:

pET28a-POPZA$_4$ and pEVOL-pAzF[1] were co-transformed into electrocompetent *E. coli* BL21 (DE3). Transformed cells were allowed to recover in SOC medium (37 °C, 50 min), then plated onto LB kanamycin + chloramphenicol agar plates (6.25 g LB powder mix, 4 g agar, 250 mL DDI water, 0.05 mg/mL kanamycin, 0.05 mg/mL chloramphenicol), and incubated at 37 °C for 16 h. Several colonies appeared on overnight-incubated plates; a single colony from this plate was inoculated in 5 mL 2YT medium having antibiotics with the same concentrations as above. The culture was incubated overnight at 37 °C with constant shaking at 250 rpm. On the following day, 5 mL of the overnight cultures was used to inoculate 500 mL of fresh 2YT media having the same antibiotics, in 5 L Erlenmeyer flask. The culture was incubated at 37 °C, 250 rpm, and protein expression was induced by adding 1mM IPTG, 2mM 4-Azido-phenyl alanine and 1% (w/v) L-arabinose when OD_{600} reached 1. The induced culture was allowed to grow for 12 hours, and then the cells were harvested by centrifugation at 4 °C, 3000 x g for 20 minutes. Cell pellets were re-suspended in 30 mL PBS (pH 7.5) and sonicated (40 amplitude, 30 second burst, 10 minute total process). Lysed culture was clarified by centrifugation at 16000 x g, 4 °C for 30 minutes and supernatant thus obtained

was purified by Ni-NTA resin using manufacturer's instructions. Purified protein was buffer exchanged to 10 mM Tris (pH 7.5) and measured by Pierce® BCA Protein Assay Kit as recommended.

Synthesis of aryldiazoacetates and cyclopropanes:

Entry	R^1	R^2	R^3	Diazo	cyclopropane
1	H	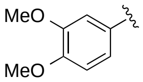	Me	**2a**	**3a**
2	H	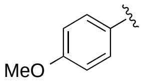	Me	**2b**	**3b**
3	H	Ph	Me	**2c**	**3c**
4	H	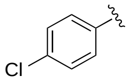	Me	**2d**	**3d**
5	H	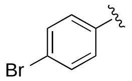	Me	**2e**	**3e**
6	H	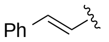	Me	**2f**	**3f**
7	H	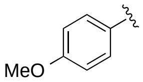	Et	**2g**	**3g**
8	OMe	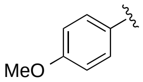	Me	**2b**	**3h**
9	Cl	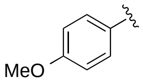	Me	**2b**	**3i**

General procedure for the synthesis of aryldiazoacetates[4]*:*

The arylacetate (3 mmol, 1 equiv), *p*-ABSA (1.3 equiv), 20 mL acetonitrile were added to a 100-mL round-bottom flask with a magnetic stir bar. The reaction mixture was cooled to 0 °C using an ice bath under nitrogen. 1,8-Diazabicycloundec-7-ene (DBU, 1.4 equiv) was then added to the stirring mixture over the duration of 5 min. After the addition of the DBU, the

reaction mixture was stirred at 0 °C for an additional 30 min. The ice bath was removed and the reaction mixture was stirred for 24 h at room temperature. The resulting solution was quenched with saturated NH_4Cl solution and the aqueous layer was extracted with diethyl ether (3 x 50 mL). The organic extracts were combined, washed with H_2O, and dried over $MgSO_4$. The organic layer was then concentrated under reduced pressure and purified using silica gel column chromatography (10:1 hexanes/EtOAc). Diazoacetates **2a-f**[5, 6] were synthesized according to the general procedure and characterization match previous literature.

Ethyl 4-methoxyphenyldiazoacetate (2g): Title compound was prepared by the general procedure and obtained as an orange solid with 47% yield. ^{1}H NMR (500 MHz; $CDCl_3$) δ 7.38 (d, *J* = 9.0 Hz, 2H), 6.94 (d, *J* = 9.0 Hz, 2H), 4.32 (q, *J* = 7.1 Hz, 2H), 3.81 (s, 3H), 1.33 (t, *J* = 7.1 Hz, 3H); ^{13}C NMR (125 MHz, $CDCl_3$) δ 165.9, 158.2, 126.1, 117.2, 114.7, 61.0, 55.5, 14.6 ($C=N_2$ signal missing); IR (KBr, cm^{-1}): 2086.2, 1700.4. HRMS (ESI-MS) calcd for $C_{11}H_{13}O_3$ $(M-N_2+H)^+$ 193.0865, found 193.0868.

General procedure for the synthesis of cyclopropanes with $Rh_2(R\text{-}DOSP)_4$[4]*:*

Styrene (5 equiv) and Rh_2(R-DOSP)$_4$ (0.01 equiv) were added to a 25-mL round bottom flask (flask A) equipped with a magnetic stir bar and degassed using vacuum/nitrogen cycles (x3). 3 mL pentane was added to flask A under nitrogen. The aryldiazoacetate (0.5 mmol, 1 equiv) was added to a separate 25-mL round bottom flask (flask B) and degassed using vacuum/nitrogen cycles (x3). 5 mL pentane was added to flask B under nitrogen. The

contents in flask B were then added to flask A using a syringe pump for the duration of 1 h. After the addition, the reaction mixture was stirred for one additional hour. The reaction mixture was concentrated under reduced pressure and purified using silica gel column chromatography (increasing gradient starting at 10:1 hexanes/EtOAc). Cyclopropanes (**3a-f**, **3h-i**)[4, 7] were synthesized according to the general procedure and characterization match previous literature.

(1*S*,2*R*)-ethyl 1-(4-methoxyphenyl)-2-phenylcyclopropanecarboxylate (3g): Title compound was prepared by the general procedure and obtained as a white solid with 58% yield. ^{1}H NMR (500 MHz; $CDCl_3$) δ 7.06-6.92 (m, 5H), 6.77 (m, 2H), 6.65 (d, *J* = 8.8 Hz, 2H), 4.12 (m, 2H), 3.72 (s, 3H), 3.05 (dd, *J* = 9.2 and 7.2 Hz, 1H), 2.11 (dd, *J* = 9.3 and 4.8 Hz, 1H), 1.81 (dd, *J* = 7.2 and 4.8 Hz, 1H), 1.18 (t, *J* = 7.2 Hz, 3H); ^{13}C NMR (125 MHz, $CDCl_3$) δ 174.1, 158.5, 136.8, 133.0, 128.2, 127.8, 127.1, 126.3, 113.2, 61.3, 55.2, 37.0, 33.0, 20.5, 14.3; HRMS (ESI-MS) calcd for $C_{19}H_{20}O_3$ $(M+H)^+$ 297.1491, found 297.1495.

Product **4** was prepared with previous reported methods[8].

NMR Spectra

Compound **2g**

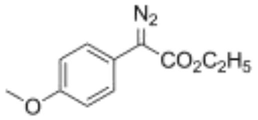

^{1}H NMR spectrum of **2g**

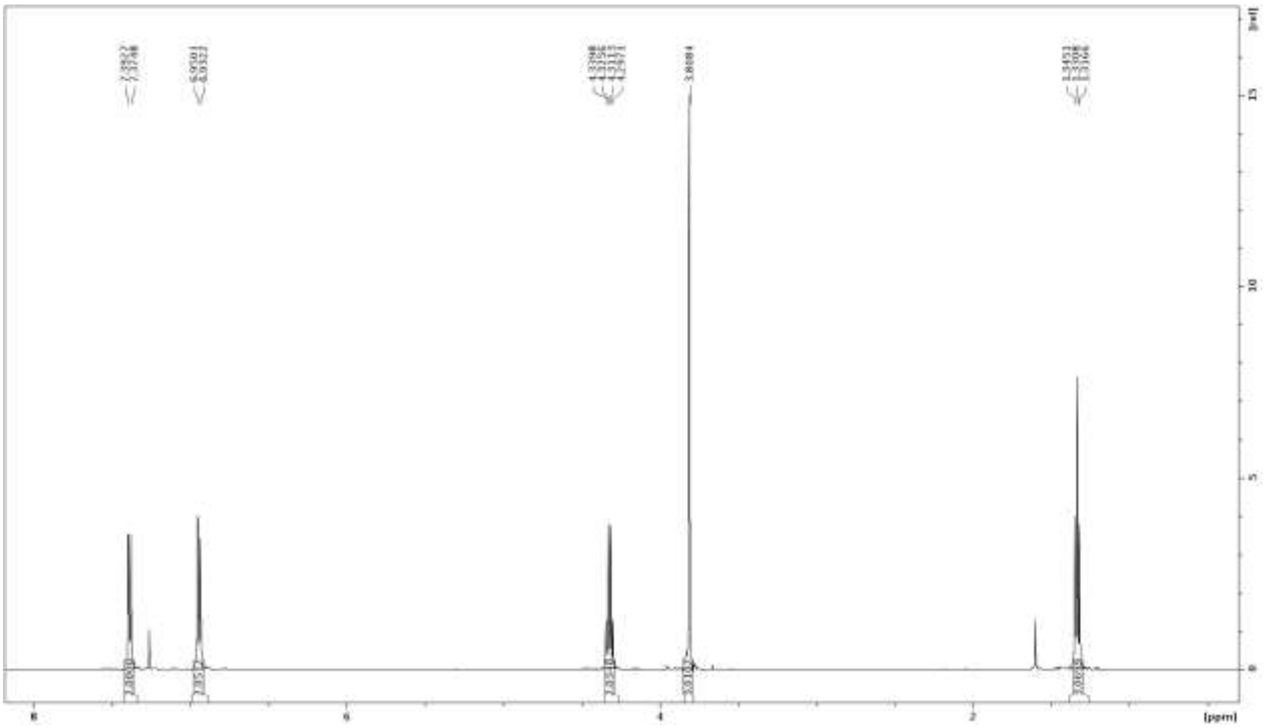

^{13}C NMR spectrum of **2g**

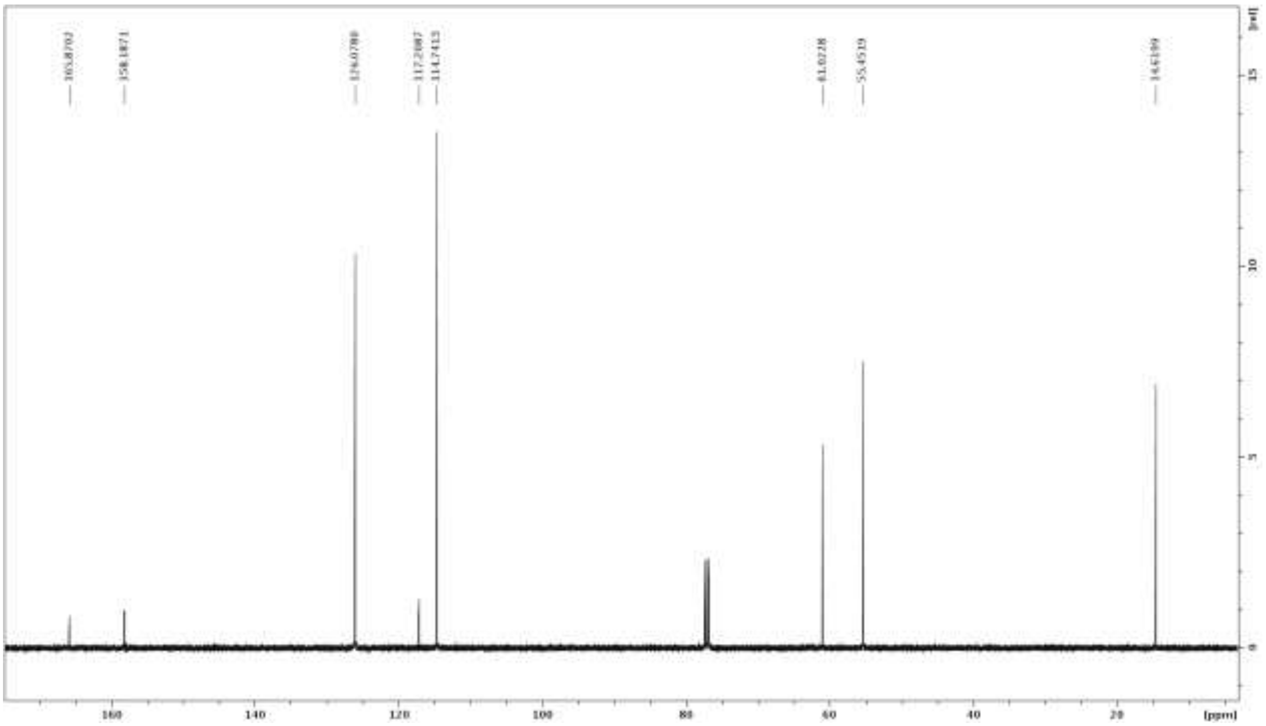

Compound **3g**

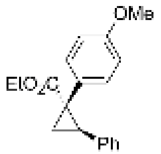

^{1}H NMR spectrum of **3g**

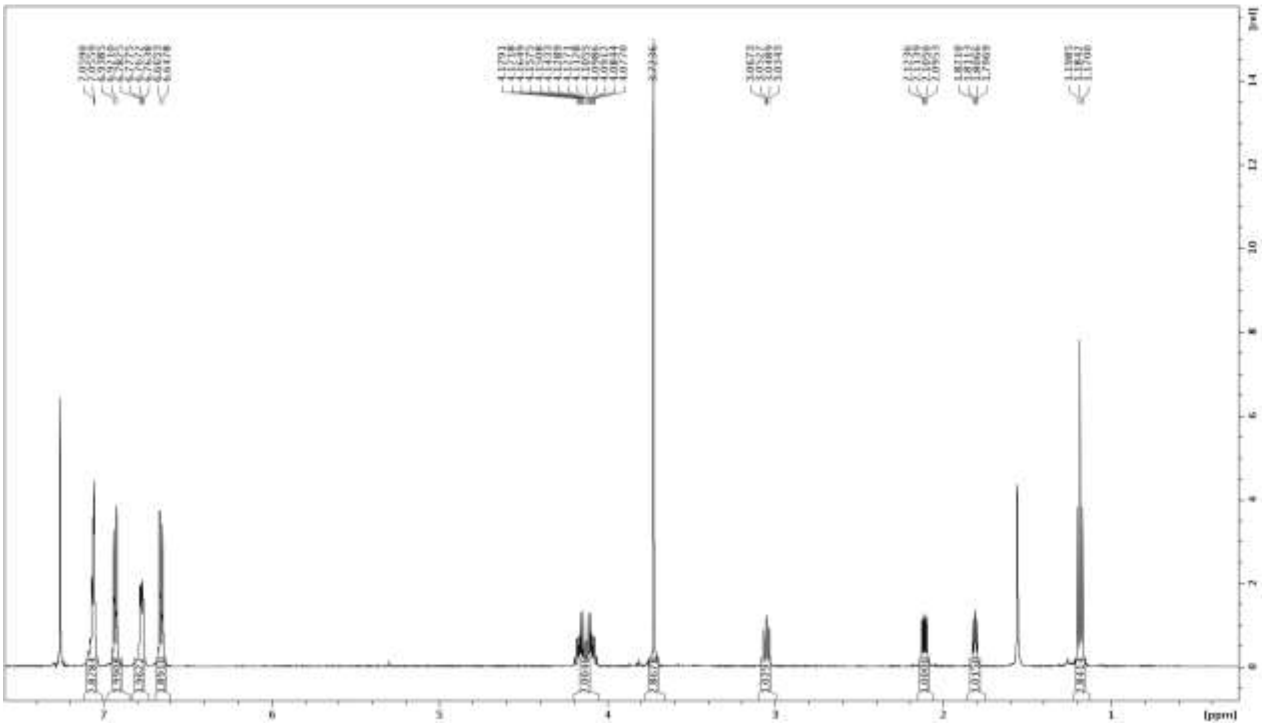

^{13}C NMR spectrum of **3g**

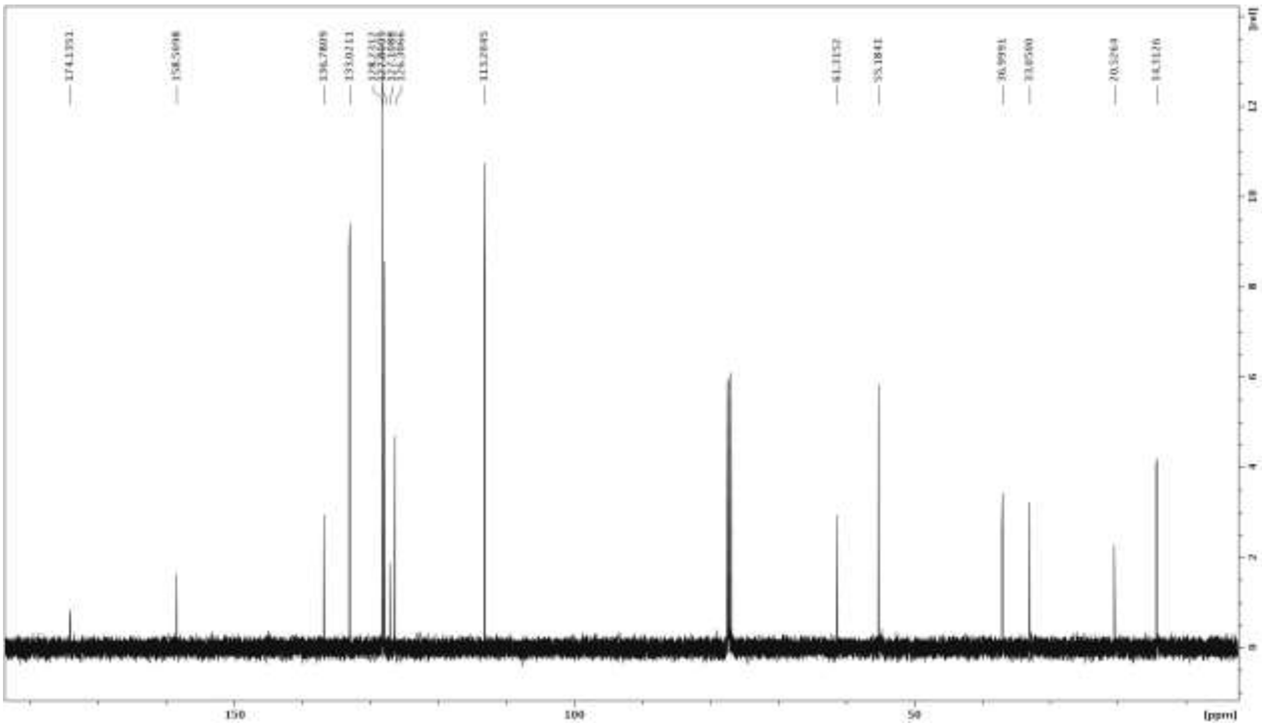

Preparation of Artificial Metalloenzymes (bioconjugation):

A solution of the POP-Z mutant (480 µL, 75 µM in 50 mM Tris-HCl buffer, pH 7.4) and a solution of cofactor **1** (120 µL, 0.75 mM in ACN, 0.655 mg/mL) were added to a 1.5 mL microcentrifuge tube and shaken at 750 rpm at 4 °C overnight. The final concentrations were: 60 µM POP, 150 µM **1**, 20 vol% acetonitrile/Tris buffer. The resulting solution was treated with 100 µL azide agarose resin, and rotated on the Labquake™ Tube Shaker/Rotator in a 4 °C cold cabinet for 24 h to remove excess cofactor. The suspension was then centrifuged at 5000 rpm for 3 min and the supernatant was transferred to a new microcentrifuge tube. The resin was rinsed twice with 600 µL 50 mM Tris-HCl buffer and centrifuged at 5000 rpm for 3 min. These supernatants were combined with the first supernatant and buffer exchanged to proper buffers for use in biocatalysis or characterization. ESI-MS were used to characterize the bioconjugates. It is worth noting that the bioconjugation reaction often does not go to completion (40 % ~ 60 % incorporation of the dirhodium cofactor was typically observed), depending on specific mutations in the POP scaffold, based on high resolution ESI-MS. This results in part from reduction of azide to anline as indicated by HR ESI-MS, although we did not observe this process in our earlier work.[3] Because of this, the effective ArM concentration was determined according to the following method: the total protein concentration was calculated based on its absorbance at 280 nm (A_{280}) and the calculated extinction coefficient for the protein (109,210 $M^{-1}cm^{-1}$ from ExPASy), which is consistent with concentrations measured by Pierce® BCA Protein Assay Kit; the cofactor absorbance at 280 nm is negligible relative to POP in aqueous solution under the concentrations used; the efficiency of dirhodium

incorporation was calculated based on the ratio of the high resolution ESI-MS peak intensity of the ArM and scaffold ($I_{ArM}/(I_{ArM}+I_{scaffold})$); the effective ArM concentration was calculated by multiplying the total protein concentration by the efficiency of dirhodium incorporation ([ArM]=[Total protein]*($I_{ArM}/(I_{ArM}+I_{scaffold})$). This method assumes that both the scaffold and the ArM possess similar response factors in the mass spectrometer. The effective ArM loading was adjusted to 1 mol% with respect to the dirhodium cofactor in bioconversions and 0.5 % with respect to the dirhodium cofactor in kinetic study.

MS Characterization of POP metalloenzyme:

For ESI-TOF MS analysis, a sample of protein was desalted with centrifugal filters to a mixture of water: acetonitrile: glacial acetic acid (49.5: 49.5: 1, v/v). The final protein concentration was 50 μM. Acquisition of the spectra was perfomed by flow injection analysis with fragmenter set at 100V-200V. Raw ESI spectra (shown in Supplementary Figure 1) were deconvoluted using the Agilent Chemstation LC/MSD data deconvolution module. The deconvoluted masses are in good agreement with the predicted masses (Supplementary Table 2). It should be noted that the mass for POP- ZA_4-HFF is typically observed 20-30 Da lower than its theoretical mass, consistent with putative loss of N_2 from the azide.

Bioconversion:

Solutions of aryldiazoacetate (25 μL, 96 mM, in THF), styrene (25 μL, 485 mM, in THF),

and POP-ZA$_4$-X-**1** solution (500 µL, the effective ArM concentration adjusted to 48 µM with respect to the dirhodium cofactor according to the aforementioned method) were added to a 1.5 mL microcentrifuge tube. The final concentrations of the reagents were: 22 mM olefin, 4.4 mM aryldiazoacetate, 44 µM POP- ZA$_4$-X-**1**. The resulting mixture was left shaking at 750 rpm at 4 °C overnight. The reaction was quenched by adding 20 µL 1,2,4-trimethoxybenzene solution (30 mM, in THF) and 600 µL ethyl acetate. The mixture was vortexed and centrifuged (15,000 x g, 3 min). The top organic layer was collected and the bottom aqueous layer was extracted with 600 µL ethyl acetate twice. The organic extracts were combined, evaporated and re-dissolved in 200 µL THF. 4 µL THF solution of the crude product was analyzed on RP-HPLC to determine conversions; 50 µL THF solution of the crude product was purified on preparative-HPLC to isolate the cyclopropane product, which was analyzed on NP-HPLC to determine enantioselectivities. The conversions and enantioselectivities were reported as the average of two trials from the same batch of ArM set up in parallel. The RP-HPLC to determine conversions was performed on an Agilent 1100 Series HPLC system using an Agilent Eclipse Plus C18 column (95 Å, 3.5 µM, 4.6 mm i.d. x 150 mm), with with a flow rate of 1.0 mL/min and detection wavelength set at 230 nm. The following gradient was used: 10 % to 70 % B from 0-10 min, 70 % B from 10-15 min, 70 % to 100 % B from 15-18 min, 100 % B from 18-22 min, 4 min post-run (solvent A: water containing 0.1% TFA; solvent B: CH_3CN). The preparative HPLC used the same method as above. The NP-HPLC to determine enantioselectivities was performed on Agilent 1100 Series HPLC system using a Phenomenex Lux® 3u Cellulose-1 column (1000 Å, 3 µM, 4.6 mm i.d. x 250 mm), with a flow rate of 1.0 mL/min and detection wavelength set at 230 nm.

Representative traces of chiral-HPLC for determining enantioselectivities in bioconversions are shown in Supplementary Figure 2. Because the dirhodium cofactor concentration in ArM was normalized as described above, the batch-to-batch variations in the catalytic experiments were minute (<1% for ee and <5% for conversion), regardless of bioconjugation conversion, as shown in Supplementary Table 3.

Qualitative Kinetic Analysis:

The conditions used in bioconversions were slightly modified for kinetic experiment. In a 1.5 mL microcentrifuge tube, a solution of **2** (12.5 µL, 96 mM, in THF), a styrene solution (12.5 µL, 485 mM, in THF) and POP-ZA_4-X-**1** solution (250 µL, the effective ArM concentration adjusted to 24 µM with respect to the dirhodium cofactor according to the aforementioned method) were added. The resulting mixture was left shaking at 750 rpm at 4 °C. The final concentrations of the reagents were: 22 mM styrene, 4.4 mM **2**, 22 µM POP- ZA_4-X-**1**.

To determine conversion of product (**3**), the following workup was used: after the set time, the reaction was quenched by adding 30 µL 1,3-dimethoxybenzene solution (30 mM, in THF) and 1000 µL ethyl acetate. The mixture was vortexed and centrifuged (15,000 x g, 3 min). The top organic layer was analyzed by RP-HPLC. The conversions at all the time points were reported as the average of two trials from the same batch of ArM set up in parallel. The RP-HPLC to determine conversions was performed on an Agilent 1290 Series HPLC system using an Agilent Eclipse Plus C18 RRHD column (300 Å, 1.8 µM, 2.1 mm i.d. x 50 mm), with with a flow rate of 0.4 mL/min and detection wavelength set at 230 nm. The following gradient was used: 60 % B from 0-5 min, 60 % to 100 % from 5-7 min, 100 % from 7-8 min,

100 % from 8-8.5 min, 1.5 min post-run (solvent A: water containing 0.1% TFA; solvent B: CH_3CN). The data are shown in Supplementary Table 4 and Supplementary Figure 3.

To determine conversions of **2, 3, 4**, a slightly different workup was used: after the set time, the reaction was quenched by adding 30 µL 1,3-dimethoxybenzene solution (30 mM, in THF) and 400 µL dichloromethane. The mixture was vortexed and centrifuged (15,000 x g, 3 min). The organic layer was collected and the aqueous layer was extracted twice with 400 µL dichloromethane. The organic layer was combined and analyzed by RP-HPLC. The conversions at all the time points were reported as the average of two trials from the same batch of ArM set up in parallel. The RP-HPLC to determine conversions was performed on an Agilent 1290 Series HPLC system using an Agilent Eclipse Plus C18 column (95 Å, 3.5 µM, 4.6 mm i.d. x 150 mm), with with a flow rate of 1 mL/min and detection wavelength set at 230 nm. The following gradient was used: 10 % to 73 % from 0-7 min, 73 % from 7-10 min, 73 % to 100 % from 10-12 min, 4 min post-run (solvent A: water containing 0.1% TFA; solvent B: CH_3CN). The data are shown below in Supplementary Table 5, 6 and Supplementary Figure 4/5.

*Additional kinetic experiment for **5***:

Solutions of **2** (12.5 µL, 96 mM, in THF), styrene (12.5 µL, 485 mM, in THF), and POP-ZA_4-X-**1** (250 µL, 24 µM) were added to a 1.5 mL microcentrifuge tube. The resulting mixture was left shaking at 750 rpm at 4 °C. At 30-min and 60-min time points, solutions of additional **2** and styrene were added (**2:** 12.5 µL, 96 mM, in THF; styrene: 12.5 µL, 485 mM,

in THF). The following workup was used: after the set time, the reaction was quenched by adding 30 µL 1,3-dimethoxybenzene solution (30 mM, in THF) and 1000 µL ethyl acetate. The mixture was vortexed and centrifuged (15,000 x g, 3 min). The top organic layer was analyzed by RP-HPLC. The conversions at all the time points were reported as the average of two trials from the same batch of ArM set up in parallel. The data are shown below in Supplementary Table 7 and Supplementary Figure 6.

Circular Dichroism (CD) Analysis

CD spectra were acquired using a 10 mm pathlength quartz cuvette. All spectra were acquired at 25°C. Protein concentration was fixed at 10 uM (determined by A_{280}) in 100 mM sodium phosphate buffer pH 7.0.

Temperature stability profiles were acquired at 10 uM protein concentration in 100 mM sodium phosphate buf fer pH 7.0. CD curves were acquired at 10°C intervals from 50°C to 100°C, with a heating gradient of 2°C/min. Acquisition was commenced after samples were equilibrated for 2 minutes at each temperature step. Shown in Supplementary Figure 7 is the temperature stability profile for wild-type POP.

References:

[1] Young, T. S., Ahmad, I., Yin, J. A. & Schultz, P. G. An enhanced system for unnatural amino acid mutagenesis in E. coli. *J. Mol. Biol.* **395,** 361–374 (2010).

[2] Heckman, K. L. & Pease, L. R. Gene splicing and mutagenesis by PCR-driven overlap extension. *Nat. Protoc.* **2,** 924-932 (2007).

[3] Yang, H., Srivastava, P., Zhang, C. & Lewis, J. C. A general method for artificial metalloenzyme formation through strain-promoted azide–alkyne cycloaddition. *Chembiochem*. **15,** 223-227 (2014).

[4] Chepiga, K. M. *et al*. Guide to enantioselective dirhodium(II)-catalyzed cyclopropanation with aryldiazoacetates. *Tetrahedron.* **69,** 5765–5771 (2013).

[5] Davies, H. M. L. & Antoulinakis, E. G. Intermolecular metal-catalyzed carbenoid cyclopropanations. *Org. React.* **57,** 1-313 (2001).

[6] Davies, H. M. L., Hansen, T. & Churchill, M. R. Catalytic asymmetric C−H activation of alkanes and tetrahydrofuran. *J. Am. Chem. Soc.* **122,** 3063-3070 (2000).

[7] Panaro, S. A. & Davies, H. M. L. Effect of Rhodium carbenoid structure on cyclopropanation chemoselectivity. *Tetrahedron*. **56,** 4871-4880 (2000).

[8] Chen, P. & Yang, W. Kinetic resolution of mandelate esters via stereoselective acylation catalyzed by lipase PS-30. *Tetrahedron Lett*. **55,** 2290-2294 (2014).
